# Asymmetric mucosal structure, mesenteric versus antimesenteric, in mouse, rat, and human small intestines

**DOI:** 10.14814/phy2.15547

**Published:** 2022-12-21

**Authors:** Anna Casselbrant, Herbert F. Helander

**Affiliations:** ^1^ Department of Surgery, Institute of Clinical Sciences Sahlgrenska Academy at the University of Gothenburg Gothenburg Sweden

**Keywords:** anti, human, jejunum, mesenteric, morphometry, mucosa, villi

## Abstract

The morphology of the small intestinal mucosa is reflected by the degree of stimuli. Previous studies have come to different conclusion about whether the mucosa is equally symmetrical. The aim of the study is to investigate whether there are structural differences in the mesenteric versus antimesenteric mucosa in mice, rats, and humans. Jejunal biopsies from mice and rats were saved. Samples from human small intestine were obtained from patients undergoing Roux‐en‐Y gastric bypass surgery. Fixed samples were used to morphologically evaluate villus height and enlargement factor due to villi. The number of goblet cells, mast cells, enteroendocrine cells, and Paneth cells were histologically analyzed in the villus structure. Cell turnover was analyzed by Ki‐67 staining. There was a significant increased villi height and villus enlargement factor antimesenterically in mice, rats, and human small intestines. The distribution of goblet cells, mast cells, and Paneth cells were equal while the number of enteroendocrine cells was increased antimesenteric in the human samples. The crypt mitotic activity was almost 20% higher in the antimesenteric part of jejunum. In summary we found longer villi, greater surface enlargement, and increased number of enteroendocrine cells as well as increased cell turnover antimesenterically. These differences may be of importance in understanding normal gastrointestinal physiology in health and disease.

## Graphical abstract

The symmetry of the digestive tract has been studied in mice, rats and humans. We found longer villi, greater surface enlargement and increased cell turnover anti‐mesenterically. In addition, we had more enteroendocrine cells in the anti‐mesenteric side. These differences may be of importance in understanding normal gastrointestinal physiology in health and disease.

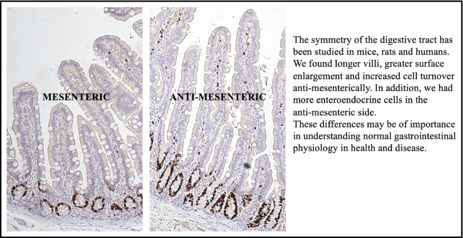

## 1 INTRODUCTION

The small intestine is composed of three parts: the duodenum, the jejunum, and the ileum. The structure of a tube, must meet the various functions such as maintaining a barrier while breaking down and absorbing nutrition's. The duodenum receives the bolus from stomach, bile and pancreatic enzymes flow into the duodenum, allow dietary digestion that are absorbed all along the length of the jejunum and ileum. The small intestine has a large surface area in relation to its luminal volume, a feature being important for effective intraluminal‐ as well as mucosa‐associated digestion and subsequent absorption of micronutrients. The total area of the digestive tract has been calculated to be about 32 m^2^ in human of which 95% belongs to the small intestinal their jejunum remains the longest part and the largest surface (Helander & Fändriks, 2014).

There is a clear proximo‐distal gradient in the nature of the crypt and villus populations of the intestinal tract (Altmann & Leblond, 1970). The symmetry of the digestive tract has been studied (Cheng et al., 1990; Meyers, 1976; Pekas, 1986). The mesenteric and antimesenteric portions of the intestinal epithelium may well be exposed to different influences affecting epithelial growth rates and the dimension of crypts and villi. In a previous study on pigs, differences were found in the jejunum and ileum on the opposite plane, mesenteric versus antimesenteric, shows that the total epithelia were significantly larger in the antimesenterial side (Pekas, 1986). However, in a study on mice, the mean crypt and villus size appeared identical at the antimesenteric side compared the mesenteric side (Cheng et al., 1990). Whether the mesenteric and the antimesenteric sides are equivalent in the human gut is lacking. The aim of this study is to investigate whether there are structural differences in the mesenteric versus antimesenteric mucosa in mice, rats, and humans.

## 2 MATERIAL AND METHODS

### 2.1 Ethics

The animal study followed the regulations outlined by the US National Institutes of Health and the European Union, and it was reviewed and approved by the local committee of the Swedish Animal Welfare Agency. The human study was approved by the Regional Ethical Review Board in Gothenburg (dnr. 261–13) and was performed in accordance with the Declaration of Helsinki. All donors were informed verbally and in writing and signed in a consent form.

### 2.2 Mouse small intestine

**FIGURE 1:**
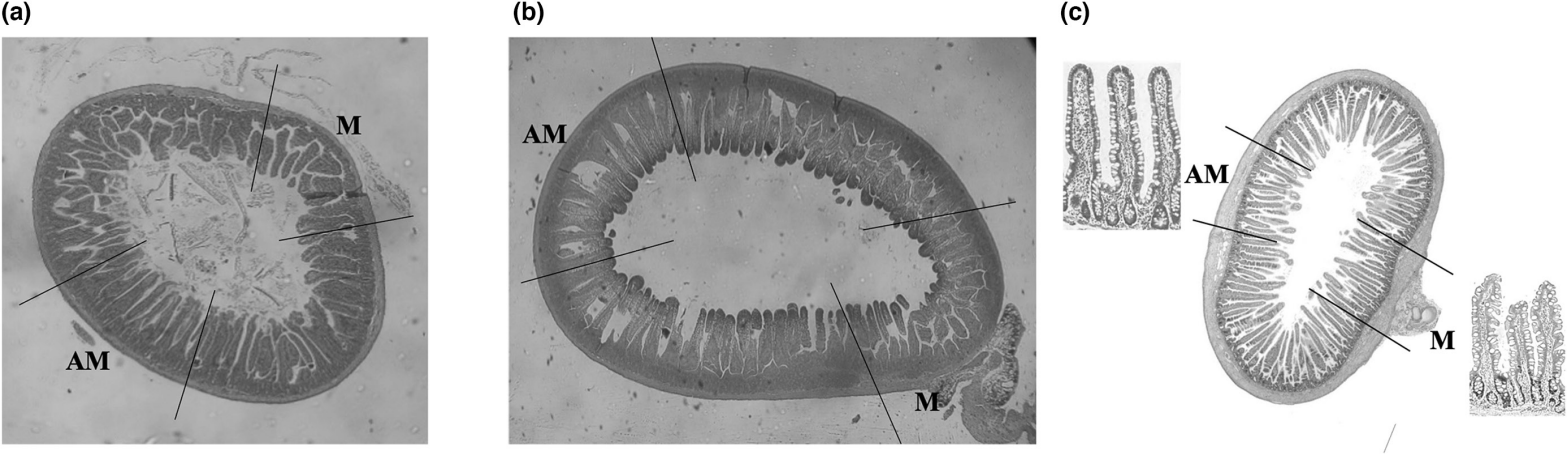
Morphological measurement of jejunal villus was performed in cross‐section from mouse (a), rat (b), and human intestines (c). The mesenteric (M) part was located and the corresponding side, that is, the antimesenteric (AM) part, was marked for measurement. Samples from the human tissue were taken during Roux‐en‐Y gastric bypass surgery where a number of smaller pieces from the mesenteric and antimesenteric section were excised before fixation.

Nine non‐fasting male mice of the C57BL6 strain, weighing 25 to 30 g were anesthetized by inhalation using isoflurane 2% to 2.5% (Forene, Abbott, Solna, Sweden). The proximal jejunum was separated from the rest of the intestine and specimens of full‐wall thickness were collected and fixed in 4% formaldehyde at the time of killing (Figure 1a). Specimens were then embedded in paraffin and stained with hematoxylin–eosin.

### 2.3 Rat small intestine

Twenty‐one non‐fasting male Sprague–Dawley rats (230–250 g) were purchased from Charles River (Sulzfeld, Germany) and acclimatized for 1 week in the university animal quarters, in 12‐h light/dark cycles, at controlled temperature, in a pathogen‐free environment, and receiving rat chow and water ad libitum. The rats were anesthetized by inhalation 2% isoflurane (Forene), the abdomen was opened, and the jejunum was rapidly resected. The specimens of full‐wall thickness were immediately placed in 4% formaldehyde, then embedded in paraffin, and stained with hematoxylin–eosin (Figure 1b).

### 2.4 Human small intestine

Six jejunal biopsies were obtained from adult patients undergoing Roux‐en‐Y gastric bypass surgery (four female, mean age 34 years, range 29–42 years) at the Sahlgrenska University Hospital, Gothenburg, Sweden. The surgical techniques included an antecolic‐antegastric Roux‐en‐Y construction with a 10–20 ml gastric pouch. The gastro‐entero‐anastomosis was constructed using a straight 45 mm stapler and complementary hand‐suturing thus creating a wide‐open gastro‐entroanastomosis. A full‐wall specimen was resected from the jejunum between the gastro‐entero and the entero‐entero anastomosis as the bowel loop was divided to create the Roux‐en‐Y construction as previously been described (Spak et al., 2010). From the circular full‐wall intestinal tract, smaller mucosal biopsies were cut from the mesenteric and the antimesenteric side (Figure 1c), immediately placed in 4% formaldehyde, then embedded in paraffin, and stained with hematoxylin–eosin.

### 2.5 Histology

Measurement of length of villi was carried out in the light microscope at ×10 times magnification. Villus length were assessed only where tissue orientation was optimal and allowed identification of tip of villus, transition from villus to crypt and the crypt bottom. We also counted the surface enlargement factor due to villi as number of intersections between grid lines and the surface of the mucosa and between the grid lines and the muscularis mucosae (Mayhew, 1991). Morphometric measurements were taken from photomicrographs of each specimen. Approximately, two samples were saved from each location from mouse and rat full‐wall samples; and on an average approximately 2 to 3 points were counted per sample in a paired fashion. From the human samples, three samples from each location were embedded and up to approximately 25 villus lengths were counting per samples in an unpaired fashion, whereas enlargement factor was measured in pairs. The microscopist (H.F.H.) was blinded with regard to the identity of the mesenteric and the antimesenteric side in the samples, until after the morphometric results had been completed.

### 2.6 The cell composition and proliferation

In the human samples, the number of goblet cells, mast cells, enteroendocrine cells, and Paneth cells were analyzed in the villus structure. To evaluate goblet cells, tissue sections were stained by periodic acidic–Schiff (PAS) staining method. Toluidine blue staining reactions were used for visibility of mast cells. Immunostaining was used for evaluate enteroendocrine cells and Paneth cells. Sections were rehydrated, antigens retrieved by boiling for 20 min in 10 mM citrate buffer (pH 6.0), blocked in 5% normal goat serum and incubated in primary antibody against chromogranin A (1:100, Abcam), and Lysozyme C (1:100, Santa Cruz Biotechnology), respectively, overnight at 4°C. After primary antibody incubation, slides were washed and incubated with secondary Alexa Fluor™ 488 antibody (Invitrogen, Stockholm, Sweden) for 2 h in darkness at room temperature. After washing, the slides were counter‐stained with Hoechst staining and cover‐slipped with ProLong Gold anti‐fade reagent (P36930, Invitrogen). Blocking buffer instead of primary antibody was used as a negative control. The immunostained slides were analyzed with a fluorescence microscope (Nikon Eclipse E400). Calculations are based on the number of positive cells in relation to mucosa size, estimated by the number of intersections of the surface epithelium using a square grid (square size 12 × 12 mm^2^).

Cell proliferation was measured by Ki‐67 in the human sample. For immunostaining, sections were rehydrated, antigens retrieved by boiling for 1 min in 50 mM borate buffer (pH 8.0), blocked in 5% normal goat serum, and incubated in primary antibody against Rb‐a‐Ki‐67 (1:100, Biocare Medical) for 1 h at room temperature. Immunoreactivity was visualized by the diaminobenzidine (DAB) method, and sections were contrasted for 30 s in Mayer's hematoxylin. Mitotic activity by use of Ki‐67 immunostaining was expressed as percent‐stained crypt cells.

### 2.7 Statistical analysis

All data are expressed as the mean ± SEM. The nonparametric Wilcoxon signed‐rank test for paired data or the nonparametric t‐test for unpaired data was used for analysis of statistical changes in the morphological analyses. All statistical analyses were performed using Prism 7 (GraphPad). Significance level was set at *p* ≤ 0.05.

## 3 RESULTS

### 3.1 Length of villi

**FIGURE 2:**
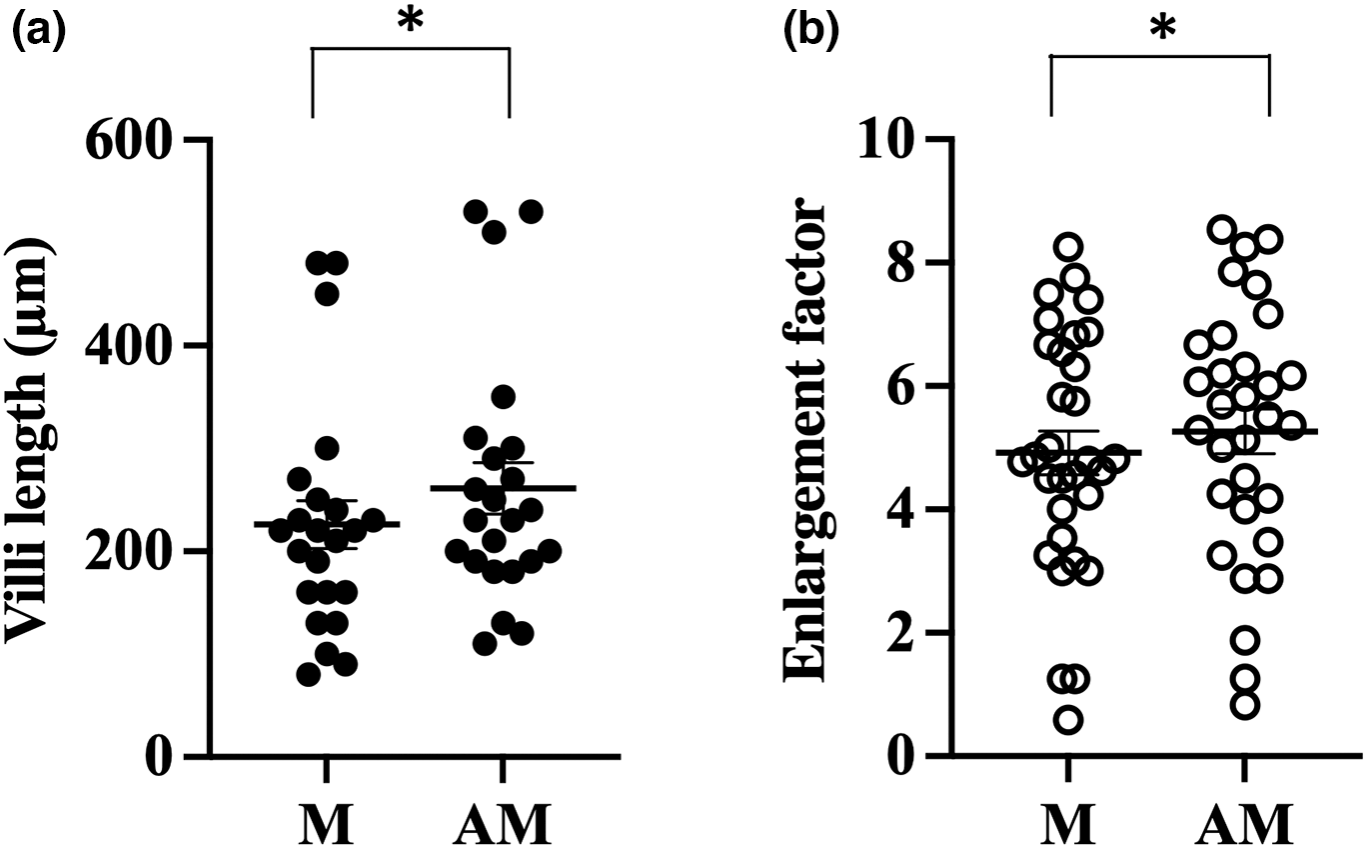
Morphological measurement of mouse small intestine. Villus length (a) and enlargement factor due to villi (b) were calculated in the mesenteric (M) and antimesenteric (AM) part of jejunum. *p ≤ 0.05 (Wilcoxon signed‐rank test).

**FIGURE 3:**
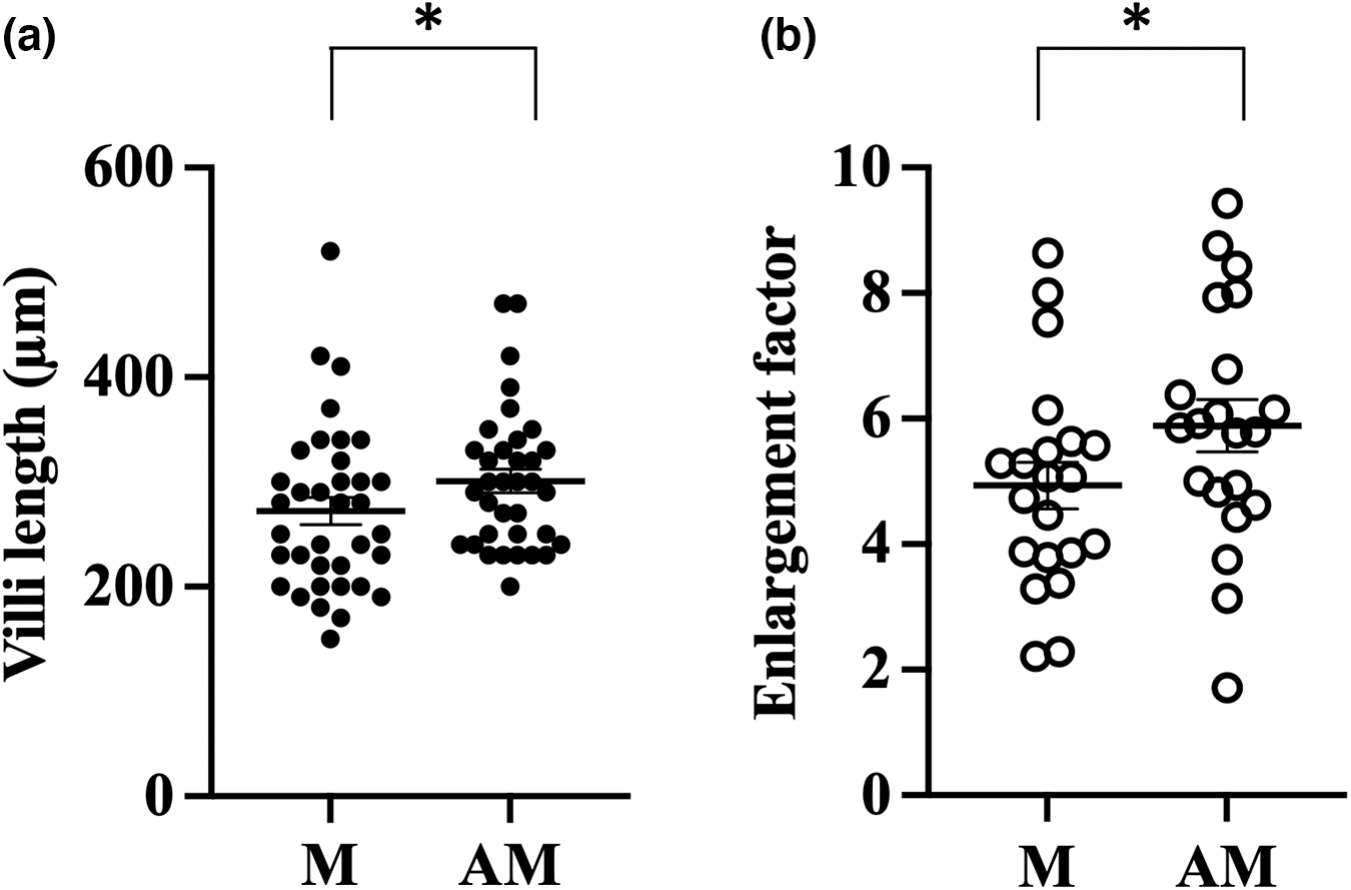
Morphological measurement of rat small intestine. Villus length (a) and enlargement factor due to villi (b) were calculated in the mesenteric (M) and antimesenteric (AM) part of jejunum. *p ≤ 0.05 (Wilcoxon signed‐rank test).

**FIGURE 4:**
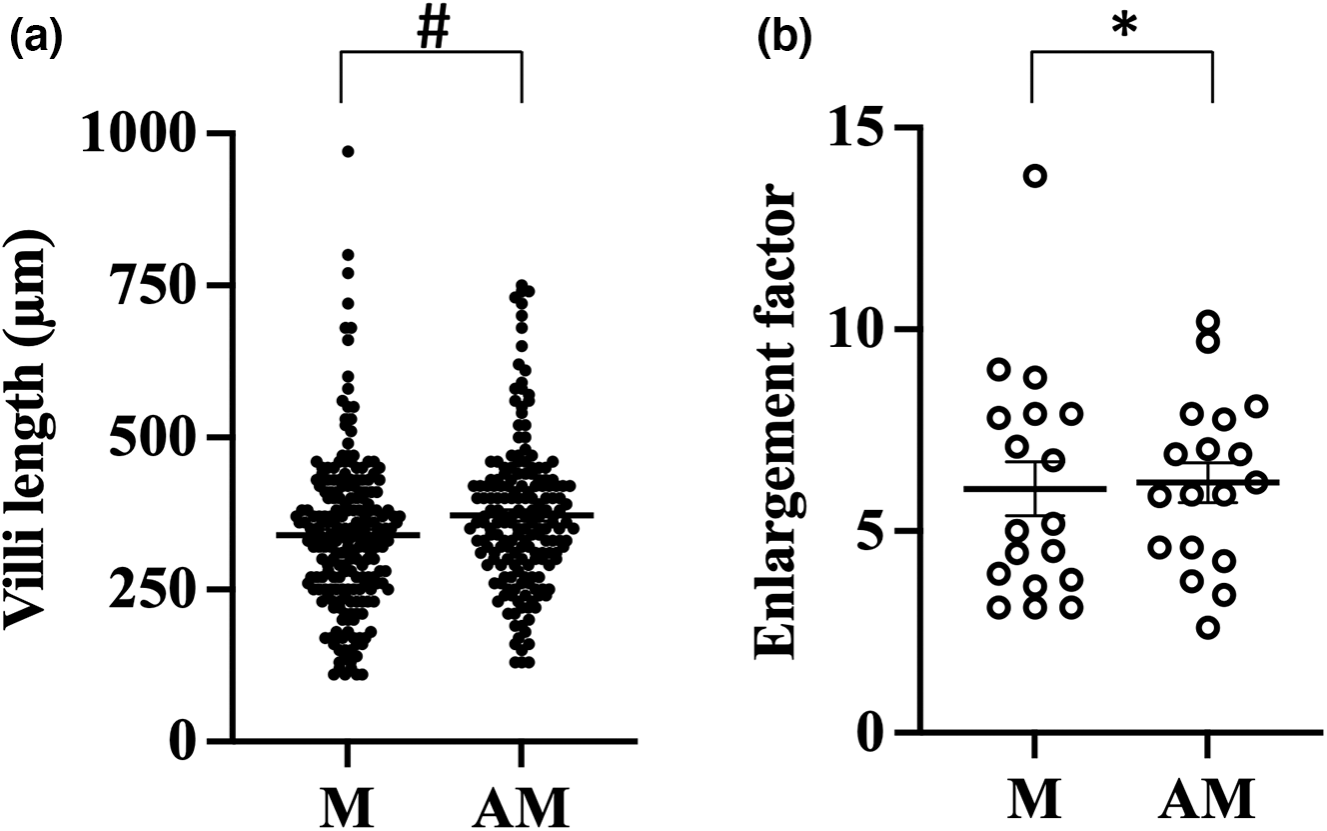
Morphological measurement of human small intestine. Villus length (a) and enlargement factor due to villi (b) were calculated in the mesenteric (M) and antimesenteric (AM) part of jejunum. #*p* ≤ 0.05 (unpaired t‐test) *p ≤ 0.05 (Wilcoxon signed‐rank test).

All morphometric data are summarized in Table 1. A total of 46 villi were measured in nine mice. The length of villi was average 226 ± 23 μm mesenteric and 261 ± 25 μm antimesenteric (*p* = 0.0006, Figure 2a). In the 21 rats, a total of 71 villi were measured and the average length was 272 ± 13 μm in the mesenteric part and 301 ± 11 μm in the antimesenteric part of the small intestine (*p* = 0.01, Figure 3a). In the six human samples, a total of 391 villi were measured. The length of villi in the mesenteric side was 400 ± 56 μm and in the antimesenteric side 420 ± 43 μm (*p* = 0.01, Figure 4a). This means that the length of the villi is about 10% longer antimesenteric in mouse, rat, and human.

**TABLE 1:** Morphometry of mice, rats, and human jejunal mucosa in the mesenterial and antimesenterial part.

	Mice		RAT		Human	
	M	AM	M	AM	M	AM
Length of villi (μm)	**226 ± 23** (*n* = 9, *N* = 23)	**261 ± 25** ^a^ (*n* = 9, *N* = 23)	**272 ± 13** (*n* = 21, *N* = 36)	**301 ± 11** ^a^ (*n* = 21, *N* = 35)	**400 ± 56** (*n* = 6, *N* = 211)	**420 ± 43** ^b^ (*n* = 6, *N* = 178)
Enlargement factor due to villi	**4.9 ± 0.4** (*n* = 9, *N* = 31)	**5.3 ± 0.4** ^a^ (*n* = 9, *N* = 31)	**4.9 ± 0.4** (*n* = 21, *N* = 21)	**5.9 ± 0.4** ^a^ (*n* = 21, *N* = 21)	**5.7 ± 0.5** (*n* = 6, *N* = 18)	**6.6 ± 0.5** ^a^ (*n* = 6, *N* = 18)

*Note*: M, mesenterial region; AM, antimesenterial region; n, number of individuals; N, number of observations. Mean ± SEM.

^a^ *p* < 0.05 M versus AM (Wilcoxon signed‐rank test).

^b^ *p* < 0.05 M versus AM (Unpaired *t*‐test).

### 3.2 Enlargement factor

The total area of the mucosal surface in relation to the relatively flat muscularis mucosae are called the surface enlargement factor and is dependent on the structure of the villi. The enlargement ratio villi/basal area mesenteric versus antimesenteric was 4.9 ± 0.4 versus 5.3 ± 0.4 in mice (*p* = 0.05, Figure 2b), 4.9 ± 0.4 versus 5.9 ± 0.4 in rat (*p* = 0.001, Figure 3b), and 5.7 ± 0.5 versus 6.6 ± 0.5 in human (*p* = 0.008, Figure 4b).

### 3.3 The cell distribution and proliferation

**FIGURE 5:**
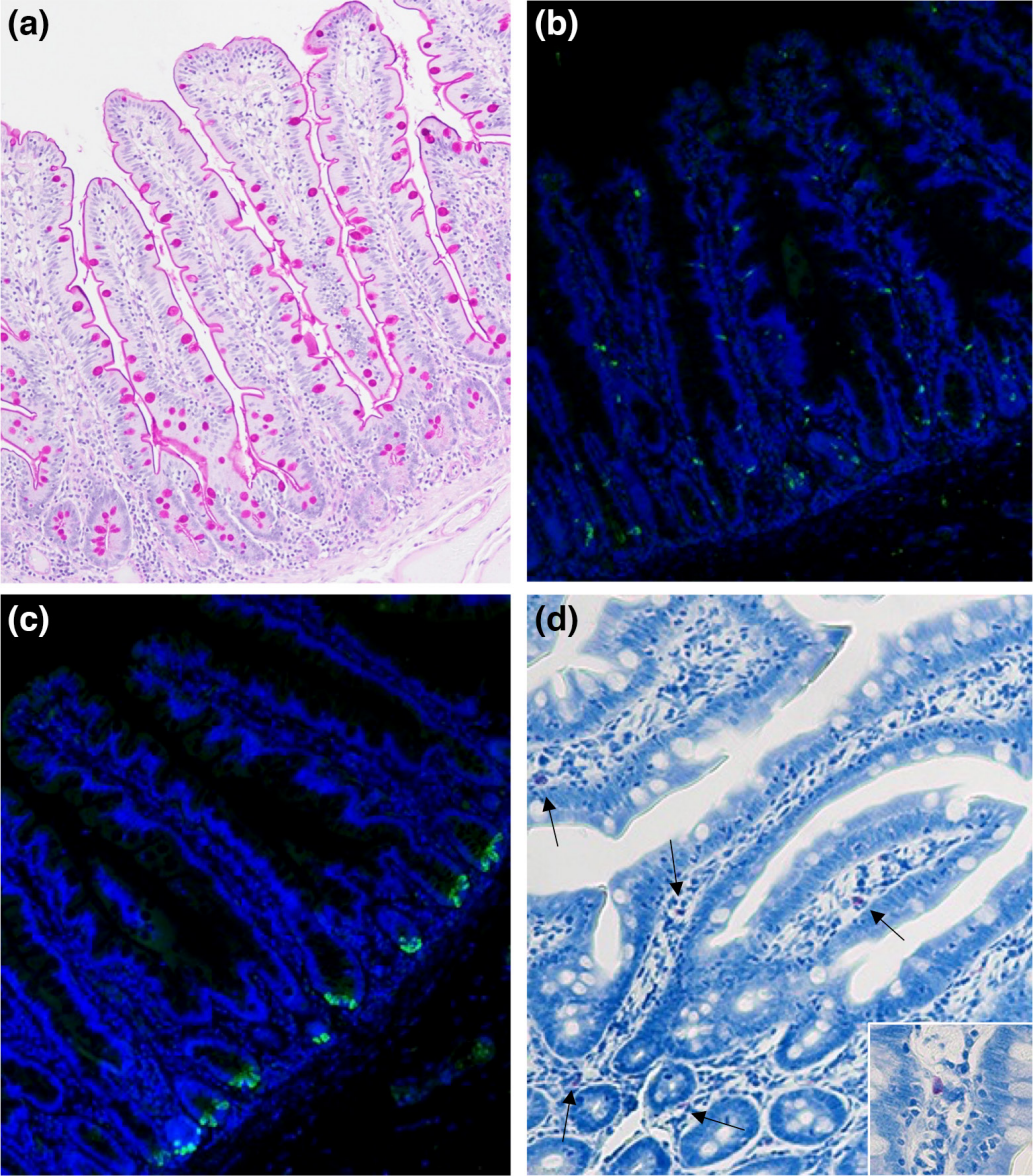
Staining of goblet cells using PAS (a), enteroendocrine cells using chromogranin A antibody (b), Paneth cells using lysozyme C antibody (c), and mast cells using toluidine blue staining (d). Arrows and inset image show stained mast cells. Original magnification x10.

**FIGURE 6:**
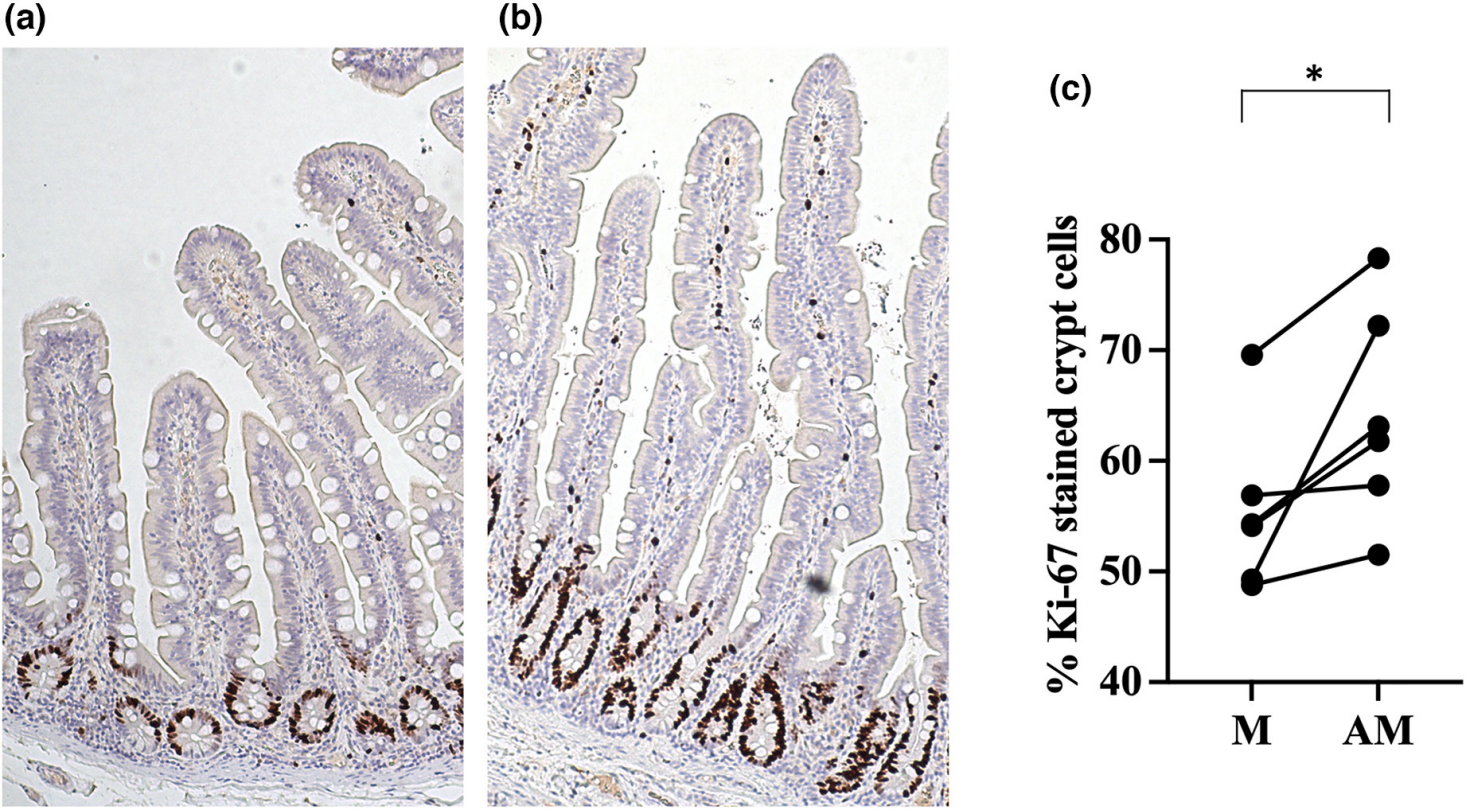
Staining for Ki‐67 in human small intestine. Panel (a) shows an example of a sectioned biopsy from the mesenteric (M) and (b) antimesenteric (AM) part of jejunum. The crypt mitotic activity was higher in the antimesenteric part of jejunum (c). *p ≤ 0.05 (Wilcoxon signed‐rank test).

The cell composition regarding goblet cells, mast cells, enteroendocrine cells, and Paneth cells was studied in the villus structure of the human samples and is reported in Table 2. The number of positive cells were statistically calculated in relation to mucosa size. The distribution of positively stained cells in the tissue is shown in Figure 5. There is no difference in the number of goblet cells, mast cells, or Paneth cells mesenterically versus antimesenterically. In contrast, the number of enteroendocrine cells was increased antimesenterically (*p* = 0.03, Figure 5b). Ki67 was used as a marker of cell proliferation. Most of the positively marked cells were found close to the bottom of the crypts of Lieberkühn, whereas in the lamina propria, only a small proportion of cells displayed immunoreactivity for Ki67. The crypt mitotic activity in the human sample was almost 20% higher in the antimesenteric part of jejunum (*p* = 0.03, Figure 6).

**TABLE 2:** The cell composition of human jejunal mucosa in the mesenterial and antimesenterial part.

	M	AM
Number of goblet cells/mucosal size or Number of goblet cells/villus	**0.74 ± 0.08** **17.4 ± 1.2** (*n* = 6, *N* = 57)	**0.71 ± 0.07** **15.7 ± 1.2** (*n* = 6, *N* = 52)
Number of enteroendocrine cells /mucosal size	**0.334 ± 0.03** (*n* = 6, *N* = 23)	**0.395 ± 0.02** ^a^ (*n* = 6, *N* = 27)
Number of Paneth cells/mucosal size	**0.23 ± 0.028** (*n* = 6, *N* = 22)	**0.25 ± 0.029** (*n* = 6, *N* = 25)
Number of mast cells/mucosal size	**0.084 ± 0.08** (*n* = 6, *N* = 23)	**0.047 ± 0.03** (*n* = 6, *N* = 27)

*Note*: M, mesenterial region; AM, antimesenterial region; n, number of individuals; N, number of observations. Mean ± SEM.

^a^ *p* < 0.05 M versus AM (Wilcoxon signed‐rank test).

## 4 DISCUSSION

This study most likely establishes that we have a morphological difference mesenteric versus antimesenteric. Villus length as well as surface enlargement factor was significantly higher antimesenteric in both the mouse, rat, and human small intestines. In addition, cell proliferation as well as the number of enteroendocrine cells was increased antimesenterically in human jejunal tissue.

Two previous studies, one on mice and one on pigs, have evaluated the difference in villus length, crypt, and/or total epithelial/mucosa (Cheng et al., 1990; Pekas, 1986). They conclude completely different results. The study on mice conclude that the mesenteric and antimesenteric villus and crypts populations are similar and they consider them together as a homogeneous population (Cheng et al., 1990). On the other hand, the study on pigs showed that there were significantly larger areas of digestive–absorptive surface and of total epithelial surface in the antimesenteric wall than in the mesenteric (Pekas, 1986). They also concluded that the tissue fraction of the muscles was larger mesenteric than antimesenteric wall.

From a functional point of view, the small intestinal mucosa is characterized by its large transport capacity with an area of about 32 m^2^ in humans (Helander & Fändriks, 2014). The surface is dependent on intestinal length, villus, and microvillus height. A previous study has seen significant increases in cell height of the epithelium and a tendency to increase surface enlargement factor due to villi in non‐fasting rats relative to fasting animals (Stenling & Helander, 1981). Animal sample used in this study were non‐fasted. The patients were, however, fasting before RYGB surgery. It seems like the mucosa has the ability to rapidly increase in size in the presence of food. More established influences that affect the mucosa are not only the pathological factors, such as bacteria and parasites, but also gluten in gluten‐intolerant individuals (Ensari & Marsh, 2018). However, studying rapid mucosal changes, or the possibility of a dynamic reason, is difficult as it probably involves the entire intestinal wall and not just the epithelial cells (Ensari & Marsh, 2018). Our study shows that we have increased cell turnover antimesenterically using Ki‐67 immunostaining, which also previous study by Grommes et al. (2013) showed in rats. Increased cell turnover is usually affected by some form of stimuli, or environmental change (Helander et al., 1990; Spak et al., 2010), but a recent study even shows that the villus height, as well as the finger‐like shape, regulates cell turnover, which can explain the increased cell turnover antimesenterically where we have longer villi (Kai, 2021).

Vagal afferent axons and terminals supplying the muscle wall are, however, more dense near the mesenteric attachment, where nerves enter the small intestine wall, and drops off progressively along the circular axis toward the antimesentery (Fox et al., 2000; Serlin & Fox, 2020; Wang & Powley, 2000). A study on premature infants shows that the neuron density of myenteric plexus is significantly higher in the mesenteric border of the small bowel compared with antimesenteric border (Nemeth et al., 2000). On the other hand, lymphoid follicles, which are important for the immune system, have been shown to lie mainly on the antimesenteric wall (Hamada et al., 2002).

Endocrine or vagal mucosal afferents respond to various stimuli, including pH, fatty acids, carbohydrates, amino acids, mechanical movements, osmo‐ and thermoreceptors, and receptors that detect bacterial products and immunogens (Egerod et al., 2018). The pattern and magnitude of innervation observed in the gut, can influence cellular activities and therefore amounts of specific cell masses or surface areas. Interestingly, in addition to an increased cell surface and villi height, we found more enteroendocrine cells antimesenterically. An important knowledge for understanding gastrointestinal functions is to investigate different stimuli and their impact on the anatomical–functional organization. Whether it is an asymmetric stimulation that is behind our findings must be evaluated in future studies. It will be a huge challenge. However, we saw no differences in the number of mucin‐secreting goblet cells, or the number of the secretory Paneth cells in the crypts of Lieberkühn, or the number of mast cells that act as first‐line immune cells in the mucosa.

Previous results show that the precision of quantitative morphometry of the small intestine is difficult and can be compromised in several ways. A complete data set must be collected and an area analysis must be carefully observed to obtain representative samples from both mesenteric and antimesenteric wall. Either the samples must be collected in a very controlled manner or many random samples are required to calculate reliable mean value. In this study, we have carefully collected a significant number of preparations from different animal species. The microscopist was completely blinded with regard to the identity of all samples. One source of error may be the degree of contraction or relaxation of the tissue at the moment of fixation, which could affect certain measurements. However, this is not very likely as the intestine has been allowed to rest in a buffer while pieces have been cut out before fixation.

In summary, this study on mice, rats, and human confirms previous results on pigs that we have longer villi, greater surface enlargement, and increased cell turnover antimesenterically. We also found an increased number of enteroendocrine cells in the antimesenteric part of the mucosa. These differences may be of importance in understanding normal gastrointestinal physiology in health and disease.

## CONFLICT OF INTEREST

The authors report no conflict of interest.
